# From Modic changes to the disc-endplate-bone marrow complex: imaging stratification, immune remodeling, and translational implications in intervertebral disc degeneration

**DOI:** 10.3389/fimmu.2026.1906279

**Published:** 2026-07-16

**Authors:** Yang Liu, Hua Huang, Maoqiang Lin, Chunwang Yang, Tao Shen, Haiyu Zhou

**Affiliations:** Department of Orthopedics, Key Laboratory of Bone and Joint Diseases of Gansu Province, The Second Hospital and Clinical Medical School, Lanzhou University, Lanzhou, Gansu, China

**Keywords:** cartilaginous endplate, DEBC classification, immune microenvironment, intervertebral disc degeneration, translational medicine, vertebral bone marrow

## Abstract

Intervertebral disc degeneration (IVDD) is frequently implicated in low back pain (LBP), yet the severity of degeneration on imaging often fails to parallel pain intensity or functional limitation. Modic changes capture abnormal marrow signal at the disc-vertebral interface, but they primarily describe subendplate marrow signal rather than lesion continuity across the disc, cartilaginous endplate, and adjacent marrow. The disc-endplate-bone marrow complex (DEBC) classification provides a complementary lesion-level imaging framework that integrates disc signal, endplate integrity, and adjacent marrow response. By incorporating short tau inversion recovery (STIR) sequences, DEBC may help identify imaging features suggestive of edema-like lesion activity; however, STIR hyperintensity should not be interpreted as direct proof of inflammation, pain generation, or a specific molecular program. This narrative and critical review synthesizes imaging, mechanistic, omics, and translational evidence to evaluate the biological plausibility and current limitations of the DEBC framework. We argue that DEBC should not replace Modic classification, assign pain causality, or guide treatment as a stand-alone criterion. Rather, its current value lies in generating testable hypotheses, improving lesion-level stratification, and supporting future imaging-to-molecular validation. Longitudinal imaging, histopathology, spatial omics, and clinical outcome studies are needed to determine whether DEBC types correspond to reproducible lesion ecologies, pain-associated phenotypes, or treatment-response patterns.

## 1 Introduction

IVDD is clinically important in part because structural degeneration on MRI and patient symptoms often diverge. Degenerative findings are common in asymptomatic individuals, whereas endplate disruption and adjacent marrow signal abnormality may be more suggestive of an interface-centered process when interpreted alongside symptoms, lesion morphology, and longitudinal change (1–7). Accordingly, disc desiccation, disc height loss, or morphological degeneration alone cannot fully explain the clinical heterogeneity of IVDD.

This imaging-symptom mismatch is central when interface lesions are interpreted. Degenerative MRI findings, including disc desiccation, disc height loss, endplate irregularity, and marrow signal changes, may be associated with pain in some populations but are also observed in asymptomatic individuals. Similarly, Modic changes have shown heterogeneous associations with LBP across cohorts and meta-analyses. Therefore, imaging phenotypes should not be interpreted as direct pain generators in isolation. Rather, they should be integrated with pain distribution, duration, functional limitation, neurological findings, competing spinal pain sources, psychosocial factors, and longitudinal clinical change.

**Figure 1:**
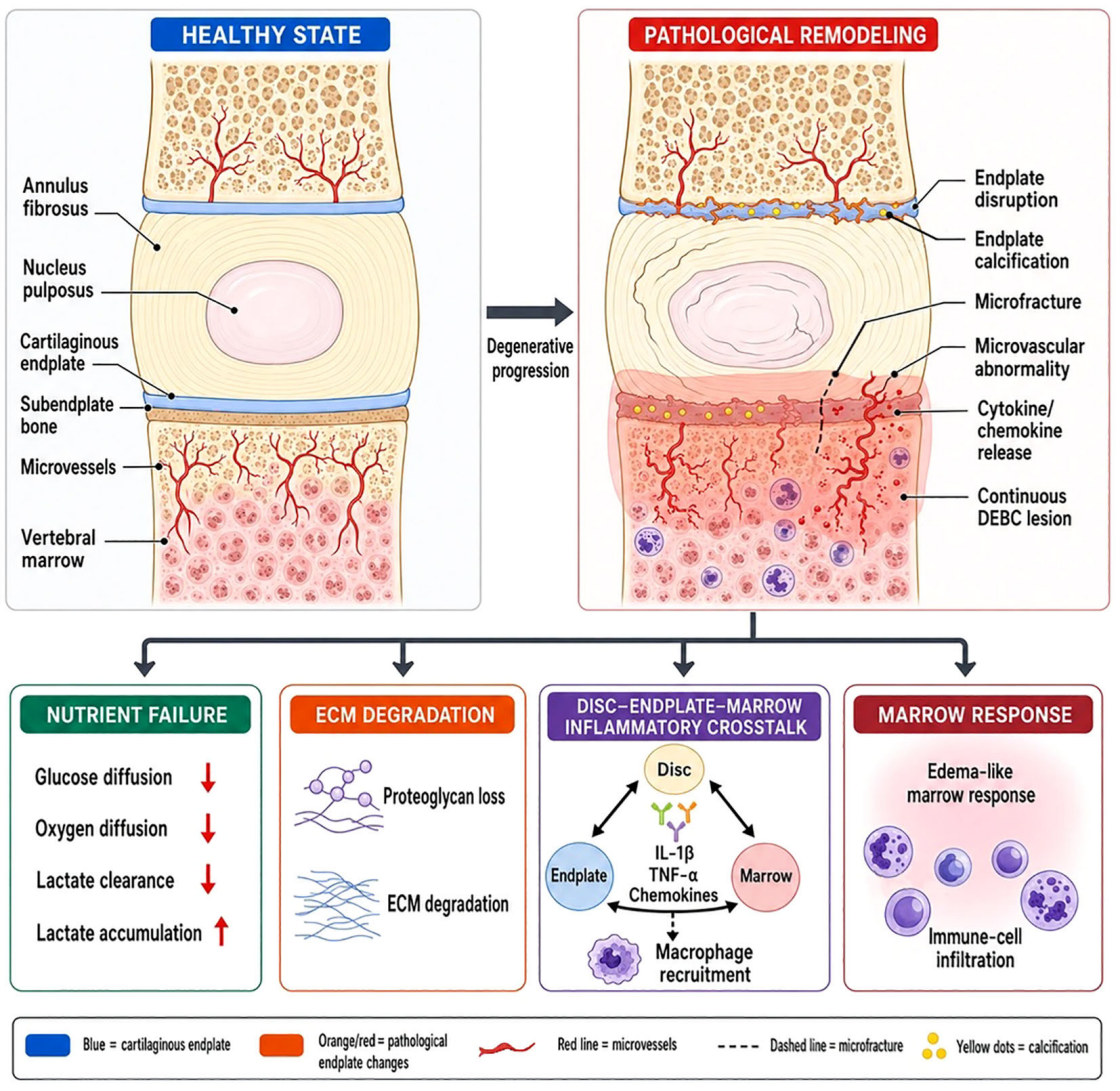
Structural basis of the disc-endplate-bone marrow complex. The schematic depicts the healthy disc-endplate-marrow interface and its transition toward pathological remodeling. In the intact state, the cartilaginous endplate supports nutrient diffusion, metabolic waste clearance, load transmission, and compartmental separation between the disc and marrow. Endplate disruption, calcification, microfracture, and microvascular abnormality may impair diffusion and barrier function, promote extracellular matrix degradation, cytokine/chemokine release, marrow edema-like response, and immune-cell infiltration, thereby establishing a continuous DEBC lesion and driving interface-centered IVDD (8, 16–18).

The disc, cartilaginous endplate, and adjacent vertebral marrow function as an integrated interface unit rather than as isolated anatomical compartments. The cartilaginous endplate mediates nutrient diffusion, metabolic waste clearance, and load transmission, and it helps maintain tissue compartmentalization between the disc and marrow (8–14). When the endplate becomes defective, calcified, or microfractured, nutritional failure, abnormal stress concentration, inflammatory mediator exchange, and marrow responses can emerge together. This pattern makes IVDD more consistent with a disease of the disc-endplate-marrow complex than with degeneration confined to the nucleus pulposus or annulus fibrosus (14–17). This interface anatomy is summarized in **Figure 1**.

This article is presented as a narrative and critical review rather than a systematic review, with the aim of synthesizing direct DEBC-related evidence and clearly distinguishing it from indirect mechanistic inference. We focus on whether DEBC can bridge Modic changes, endplate injury, immune microenvironment remodeling, and translational stratification. Rather than proposing DEBC as a replacement for Modic classification or as a stand-alone diagnostic standard, we evaluate its potential value as a complementary lesion-level framework for generating testable imaging-to-molecular hypotheses in IVDD.

## 2 Literature search and evidence interpretation

We therefore structured this article as a narrative and critical review rather than a systematic review or meta-analysis. We searched PubMed/MEDLINE, Web of Science, Embase, and Google Scholar from database inception to May 2026 using terms related to intervertebral disc degeneration, cartilaginous endplate, vertebral bone marrow, Modic changes, DEBC classification, STIR, immune microenvironment, macrophages, T cells, neutrophil extracellular traps, mast cells, single-cell or single-nucleus RNA sequencing, spatial transcriptomics, discogenic pain, and basivertebral nerve ablation. English-language original studies, imaging classification studies, mechanistic experiments, clinical studies, omics studies, and high-quality reviews were considered when directly relevant to disc-endplate-marrow interface pathology, immune remodeling, imaging stratification, or translational validation. Classic studies on Modic classification, endplate diffusion, disc nutrition, and disc immune isolation were retained when conceptually necessary. Because DEBC remains an emerging framework, direct DEBC-specific evidence was distinguished from indirect evidence derived from Modic-related, broader IVDD, animal, mechanistic, or omics studies. No formal risk-of-bias assessment, protocol registration, or meta-analysis was performed.

## 3 From Modic to DEBC: a shift in interface scale within MRI grading systems

Modic changes remain the starting point for imaging the disc-vertebral interface, but their anatomical resolution is limited. Conventional Modic classification is based mainly on T1WI and T2WI signal changes in marrow adjacent to the endplate: type 1 is generally associated with edema-like marrow response and fibrovascular remodeling and is often regarded as the Modic phenotype most compatible with active interface pathology; type 2 reflects fatty marrow replacement; and type 3 is associated with subendplate sclerosis (19, 20). Early MRI descriptions of marrow changes adjacent to degenerative endplates also support the interpretation that these signal abnormalities are closely related to the disc–endplate interface rather than being purely isolated marrow findings (21). This system is clinically accessible and widely used. However, it centers on subendplate marrow signal and does not systematically incorporate disc signal, endplate integrity, or STIR hyperintensity as an indicator of lesion activity (22, 23). In this review, lesion activity is used operationally to describe MRI features suggestive of edema-like marrow response or active remodeling, particularly STIR hyperintensity, rather than histologically proven inflammation. Thus, Modic classification can flag an abnormal interface but cannot fully characterize a continuous disc-endplate-marrow lesion.

**Figure 2:**
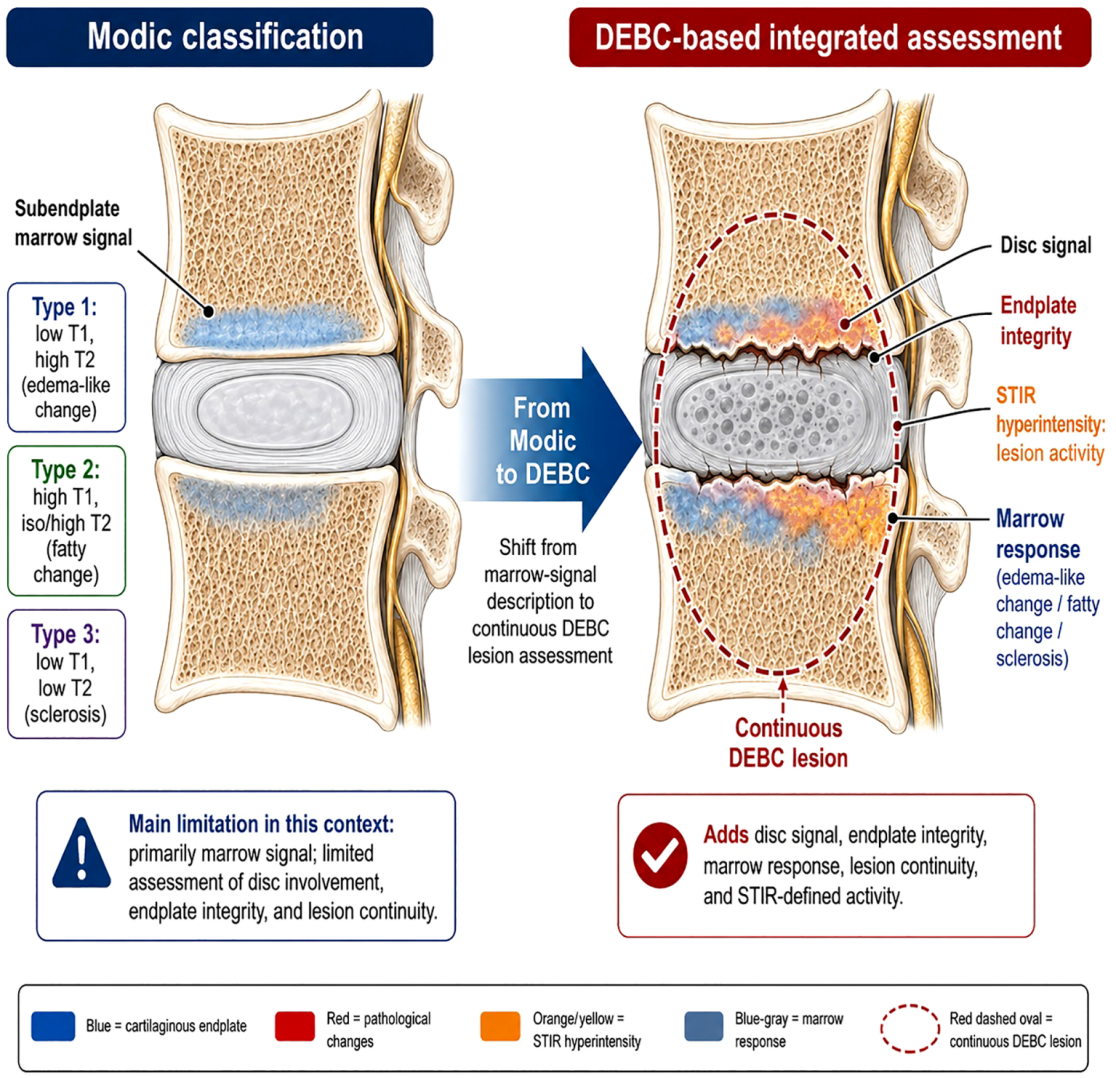
Conceptual shift from Modic classification to DEBC-based integrated assessment. Conventional Modic classification mainly characterizes subendplate marrow signal on T1WI and T2WI, whereas DEBC integrates disc signal, endplate integrity, adjacent marrow response, lesion continuity, and STIR-defined activity. This framework does not replace Modic grading; rather, it expands the assessment scale from marrow-signal description to lesion-level interpretation of the disc-endplate-marrow interface (22–25).

DEBC changes the scale at which imaging findings are interpreted. Using T1WI, T2WI, and STIR sequences, it evaluates disc signal, endplate erosion or defect, and adjacent marrow edema, fatty change, or sclerosis within the same lesion (22–24). The classification therefore serves as more than another imaging label. It places subendplate marrow signal within the context of a continuous disc-endplate-marrow lesion. The interpretive focus shifts from identifying which Modic type is present to assessing whether the DEBC lesion shows structural disruption, imaging activity, and features of remodeling. This change in imaging scale is illustrated in **Figure 2**.

Experimental work provides a complementary mechanism for the same endplate-centered argument. In a rabbit model, Feng et al. showed that physical scraping or chemical lysis of the cartilaginous endplate induced prolonged inflammatory responses in vertebral marrow and produced Modic-like MRI changes. They proposed that cartilaginous endplate injury may be one pathological source of Modic changes (25). This evidence suggests that Modic changes may be interpreted not only as marrow signal abnormalities but also as imaging consequences of endplate-driven disc-endplate-marrow lesions. The DEBC emphasis on endplate integrity and lesion continuity is therefore mechanistically plausible.

Clinical and functional imaging studies support this endplate-centered view. Cartilaginous endplate avulsion has been associated with Modic changes, endplate defects, and residual back or leg pain after lumbar discectomy. Human endplate tissue studies also link endplate defects with more severe symptoms and upregulation of the COX-2/PGE-2/EP-4 inflammatory pain axis (26–28). Together, these findings point to endplate injury as a plausible interface event connecting MRI signal change, load response, and symptom variability.

Within existing MRI grading systems, DEBC is best viewed as a complement to conventional disc degeneration grading. Pfirrmann grading and gross morphological grading remain useful for describing the degree of disc degeneration (29, 30). Esposito et al., in a scoping review of MRI-based grading systems for lumbar disc degeneration, showed that existing systems emphasize different dimensions, including nucleus pulposus signal, disc height, morphology, endplate abnormality, and adjacent marrow signal (23). This diversity highlights a practical limitation: no single imaging marker captures the structural, interface-related, and inflammatory heterogeneity of IVDD. DEBC is therefore more appropriate as a lesion-level front-end assessment used alongside Pfirrmann grade, Modic type, and clinical symptoms, rather than as a final diagnostic label.

Rajasekaran et al. proposed MRI criteria that define DEBC types according to signal pattern, endplate integrity, lesion continuity, and STIR-related activity (22). In this framework, Type A is characterized by low T1 signal, high T2 signal, and high STIR signal, a pattern compatible with edema-like marrow response and higher imaging activity. Type B shows heterogeneous high T1/T2 signal with possible partial STIR hyperintensity, which may reflect mixed edema-like and fatty components. Type C shows high T1/T2 signal with low STIR signal, a pattern more compatible with fatty change or relatively low STIR-defined activity. Type D shows low signal on T1, T2, and STIR sequences, which may correspond to sclerosis, fibrosis, or chronic low-signal structural remodeling. These interpretations should be treated as radiological hypotheses rather than as histopathological diagnoses.

DEBC and Modic types may overlap radiologically, but they are not equivalent. DEBC A/B/C/D should be interpreted as imaging phenotypes that describe lesion continuity and STIR-defined activity across the disc–endplate–marrow interface, not as fixed biological stages or a universal A-to-D disease trajectory. Their signal patterns can support clinical communication, lesion stratification, and study selection, but MRI alone cannot determine the dominant cellular program, inflammatory state, pain generator, or treatment response of an individual lesion. **Table 1** therefore summarizes the imaging features, hypothesized biological interpretations, and translational implications of each DEBC Type, while **Table 2** outlines the common overlaps and key differences between DEBC and Modic classifications.

**Table 1:** Imaging features, hypothesized biological interpretation, and translational implications of DEBC types.

Type	Main imaging features	Hypothesized biological interpretation	Translational implications
Type A	Low T1 signal, high T2 signal, and high STIR signal	Compatible with edema-like marrow response and higher STIR-defined lesion activity. Endplate disruption, inflammatory remodeling, microvascular change, or early fibrovascular response may be present, but MRI alone cannot determine the dominant pathological substrate.	May serve as a candidate imaging marker for identifying active interface lesions in longitudinal follow-up and mechanism-guided studies. It should not be used as stand-alone evidence of inflammation, pain generation, or treatment responsiveness.
Type B	Heterogeneous high T1/T2 signal with possible partial STIR hyperintensity	May reflect mixed edema-like and fatty components, chronic remodeling, or incomplete inflammatory resolution. The degree of residual activity should be interpreted together with STIR signal intensity, lesion extent, endplate integrity, symptoms, and longitudinal change.	May help stratify non-acute but potentially active interface lesions. Interpretation requires integration with clinical phenotype, functional scores, Modic status, and follow-up imaging.
Type C	High T1/T2 signal and low STIR signal	Compatible with fatty marrow change or a relatively low STIR-defined activity state. Inflammatory edema-like activity may be limited, but chronic matrix, metabolic, or marrow remodeling may persist.	More appropriate for studying low-activity or chronic remodeling phenotypes. Sensitivity to anti-inflammatory treatment should not be assumed.
Type D	Low signal on T1, T2, and STIR sequences	May correspond to sclerosis, fibrosis, or chronic low-signal structural remodeling. Lesion activity may be low, and altered mechanics, reduced transport, or subendplate bone remodeling may be more relevant than ongoing STIR-defined edema-like lesion activity.	May inform studies of chronic structural remodeling, endplate sclerosis, transport dysfunction, and regenerative failure risk. It should not be interpreted as a direct indication for a specific therapy.

DEBC types are imaging phenotypes, not definitive pathological diagnoses or treatment categories. The biological interpretations listed here are hypothesized imaging-to-molecular associations intended to guide future validation studies. STIR hyperintensity is treated as an imaging feature suggestive of edema-like lesion activity, not as direct proof of inflammation or pain generation. Validation requires MRI-targeted sampling of paired cartilaginous endplate and subendplate marrow tissues, combined with histopathology, spatial transcriptomics, multiplex immunohistochemistry, and clinical outcome correlation.

**Table 2:** Common overlap and key differences between DEBC and Modic classifications.

DEBC type	Imaging features	Common overlap with Modic type	Key difference: lesion continuity assessment
Type A	Predominantly edema-like changes with low T1 signal, high T2 signal, and high STIR signal	Usually overlaps with Modic type 1	Assesses whether disc signal, endplate defects, and adjacent marrow edema form a continuous lesion.
Type B	Mixed edema and fatty components with heterogeneous high T1/T2 signal and possible partial STIR hyperintensity	May overlap with mixed Modic type 1/2	Emphasizes residual activity suggested by STIR hyperintensity rather than fatty replacement alone.
Type C	Predominantly fatty change with high T1/T2 signal and low STIR signal	Usually overlaps with Modic type 2	Requires integration of endplate integrity and disc signal to avoid equating isolated marrow fat with a complex lesion.
Type D	Sclerotic or low-signal structural remodeling with low signal on T1, T2, and STIR sequences	May overlap with Modic type 3	Emphasizes continuity among disc low signal, endplate sclerosis, and adjacent marrow response, not marrow sclerosis alone.

This table summarizes common radiological overlap between DEBC and Modic classifications. Overlap does not imply equivalence. DEBC emphasizes continuity across the disc, endplate, and adjacent marrow, together with STIR hyperintensity as an imaging marker of lesion activity.

Cervical degeneration studies extend the DEBC concept beyond the lumbar spine but do not establish causality. Jagadish et al. reported that DEBC changes were associated with neck pain, disc herniation, and surgical treatment; the proportion of patients receiving surgery was higher when DEBC changes coexisted with disc herniation (24). These findings support the potential clinical relevance of DEBC as an imaging phenotype. However, because the evidence is mainly cross-sectional, DEBC changes cannot yet be considered a direct cause of symptoms or surgical need. These clinical and imaging observations raise a deeper mechanistic question: how does endplate injury translate into marrow response and lesion activity?

## 4 Endplate-bone marrow crosstalk: structural and molecular basis of DEBC classification

DEBC rests on the anatomical continuity and functional coupling between the cartilaginous endplate and adjacent vertebral marrow. The mechanotransduction, metabolic stress, and immune-inflammatory mechanisms discussed below should not be read as proven pathological substrates for every DEBC Type. At this stage, they are best regarded as candidate mechanisms that explain how endplate injury in IVDD may generate marrow responses, sustain lesion activity, and promote cross-tissue remodeling.

### 4.1 Structural and transport dysfunction

Endplate failure links structural damage to impaired transport and nutrient-waste exchange (31, 32, 36–39). Altered load transmission, barrier breakdown, and mechanobiological stress responses further connect endplate injury with cross-interface remodeling (33–35, 40). The cartilaginous endplate regulates nutrient diffusion and metabolic waste clearance (8–13, 31, 32), transmits mechanical load, and helps maintain tissue compartmentalization (12–14, 16). In the intact state, it limits abnormal cross-interface movement of matrix fragments, inflammatory mediators, and cellular components. Once endplate defects, calcification, microfracture, or microvascular abnormalities develop, these transport, barrier, and mechanical buffering functions can deteriorate together (14–17). Matrix mineralization and sclerotic thickening further reduce permeability, limiting oxygen, glucose, and small-solute entry into the disc while restricting lactate and catabolic waste clearance (13, 16, 36–39). Endplate injury can therefore convert a local structural defect into nutrient failure, acid metabolite accumulation, reduced matrix synthesis, and adjacent marrow response.

### 4.2 Mechanical-metabolic coupling

Mechanical overload acts on the endplate as a biological signal, not merely as a structural stressor. Piezo1 can translate membrane tension, compressive load, or matrix stiffness into Ca²^+^ influx and downstream signaling. Recent studies indicate that Piezo1 upregulation is associated with extracellular matrix (ECM) degradation, apoptosis, and degenerative progression, and that the Ca²^+^-F-actin-YAP axis can promote cartilaginous endplate degeneration and calcification (33, 34). The primary cilium-related IFT88/TRPV4 pathway may also regulate Ca²^+^ influx, ROS production, and Wnt-related pro-calcification signaling under excessive mechanical stress, thereby contributing to endplate calcification and IVDD progression (35). Taken together, these findings suggest that the endplate is not simply a load-bearing interface; it can convert abnormal mechanical cues into cellular stress, matrix remodeling, and adjacent marrow responses.

Metabolic stress provides another link between endplate transport failure and inflammatory amplification. Reduced endplate permeability restricts nutrient entry into the disc and impairs clearance of acidic metabolites, exposing nucleus pulposus and endplate cells to a chronically low-nutrient, acidic, catabolic microenvironment. Acid-sensing ion channels can promote nucleus pulposus cell inflammation and pyroptosis through the NLRP3 inflammasome (41, 42). Mitochondrial quality-control failure may further increase ROS accumulation, cellular senescence, and ECM imbalance (43). Mechanical sensing and metabolic stress should therefore be viewed as interacting processes that help maintain lesion activity after endplate injury.

Viewed as a disease loop, endplate-marrow crosstalk links structure, mechanics, metabolism, and immunity. Endplate disruption weakens diffusion and barrier function, producing substrate deprivation and impaired acid-waste clearance. The resulting low-nutrient, high-catabolic environment suppresses ECM synthesis and reduces disc load-bearing capacity. As mechanical load redistributes, abnormal stress can activate Piezo1, IFT88/TRPV4, and related pathways, leading to Ca²^+^ dysregulation, ROS accumulation, pro-calcification signaling, and cellular stress. These changes can in turn promote endplate calcification, microdamage, and marrow inflammation, creating a self-amplifying interface loop (17, 18, 33–35, 40).

### 4.3 Endplate-bone marrow inflammatory amplification

OPN-related studies bring the immune dimension of endplate-marrow crosstalk into sharper focus. Wang et al. found that osteopontin (OPN) deficiency enhanced NF-κB signaling in cartilaginous endplate chondrocytes, promoted chemokine expression, recruited macrophages, activated the NLRP3 inflammasome, and aggravated cartilaginous endplate degeneration (18). This mechanism suggests that stressed endplate cells are not passive victims of degeneration. They can actively recruit marrow immune cells through inflammatory and chemotactic signals, generating an amplification loop that links endplate cell stress, marrow immune-cell recruitment, inflammasome activation, and further endplate damage.

This immune-marrow loop helps explain why DEBC assessment should include endplate status rather than marrow signal alone. If endplate transport failure, mechanosensitive pro-calcification signaling, mitochondrial stress, and abnormal subendplate bone stiffness persist, therapies aimed only at improving the disc matrix may lack a stable interface environment (16, 17, 33, 34, 39, 43, 44). DEBC may be useful because it places endplate disruption, marrow reaction, and disc signal within the same lesion-level assessment. In doing so, it offers an imaging perspective that is closer to the putative interface loop and may help generate hypotheses about lesion activity, treatment windows, and mechanisms of therapeutic failure (22–24). The point is not to assign a single molecular pathway to each MRI type, but to treat the disc, endplate, and marrow as a connected lesion ecology that requires direct validation.

## 5 Immune microenvironment remodeling: a biological interpretation of active DEBC lesions

### 5.1 Disruption of immune isolation and cautious interpretation of the immune-pain relationship

Immune microenvironment remodeling offers one plausible biological explanation for STIR-defined activity in DEBC Type A/B lesions, but immune activity should not be equated with pain. Under physiological conditions, the avascular disc, dense ECM, and intact endplate barrier restrict immune-cell entry and preserve a relatively immune-isolated state (45–47). Annular fissures, endplate defects, and exposed matrix fragments can compromise this isolation. Once this barrier is weakened, immune-cell recruitment and inflammatory mediator diffusion may establish cross-interface communication among the disc, endplate, and adjacent marrow (18, 41, 48–52). Chemokine signaling, extracellular vesicle exchange, and immune-context-dependent paracrine interactions may further sustain this interface response (53–57). However, the presence of an imaging-active or immunologically active interface lesion does not by itself establish that the lesion is the dominant pain generator.

The imaging-pain relationship in IVDD remains probabilistic rather than deterministic. Modic changes and endplate abnormalities may increase the likelihood of pain in selected cohorts, but their association with LBP is inconsistent across studies and may be modified by age, spinal level, lesion size, chronicity, inflammatory activity, and coexisting degenerative findings (1–5, 58, 59). Pain may arise from multiple overlapping sources, including annular fissures, nerve ingrowth, basivertebral nerve-related nociception, facet joint degeneration, sacroiliac pathology, radiculopathy, muscle dysfunction, and peripheral or central sensitization (47, 60–66). Psychosocial factors and individual differences in pain modulation further weaken any one-to-one relationship between MRI signal and symptoms.

For this reason, DEBC Type A/B lesions should be interpreted as possible imaging-active interface phenotypes rather than as direct evidence of discogenic pain. Their value is more defensible as a stratification variable for mechanism-guided studies than as a diagnostic label for pain causality. Future studies should therefore correlate DEBC type with standardized pain phenotyping, functional scales, neurological examination, exclusion of competing pain generators, and longitudinal outcomes. This approach would help determine whether specific DEBC phenotypes are merely imaging descriptors, markers of biological activity, or clinically meaningful subgroups associated with pain persistence or treatment response.

### 5.2 Multicellular adaptive–innate immune networks and cytokine crosstalk

Macrophages remain the most consistently supported immune-cell hub within the IVDD inflammatory microenvironment, but active DEBC Type A/B lesions are unlikely to be sustained by macrophages alone. Earlier studies linked macrophages to inflammatory amplification, matrix degradation, and tissue remodeling; more recent reviews further suggest that macrophages modulate disc-cell activity, ECM metabolism, vascularization, and innervation (48–52, 67). Cytokines such as IL-1β, TNF-α, IL-6, and IL-17 can drive cellular stress, matrix breakdown, and inflammatory cascades, whereas NF-κB, MAPK, JAK-STAT, and NLRP3 inflammasome pathways are closely associated with apoptosis, ECM degradation, and chronic inflammatory persistence (18, 51, 68–70). These pathways echo the NF-kappaB/chemokine-macrophage recruitment-NLRP3 axis observed in OPN-deficient cartilaginous endplates, implying that endplate-marrow inflammation is maintained by a multicellular and multipathway network rather than by a single dominant signal (18).

Recent single-cell and single-nucleus sequencing studies support this broader multi-lineage model while also defining its current limits. Human degenerative endplate scRNA-seq has identified multiple chondrocyte states, macrophages, and T/NK cells, with ligand-receptor analyses suggesting macrophage-chondrocyte and T/NK cell-chondrocyte communication, including FN1- and CD74-related interactions (56). Other single-cell studies of degenerative nucleus pulposus and annulus fibrosus tissues have similarly emphasized immune-cell heterogeneity and macrophage involvement (71–75), whereas later datasets further support inflammation-associated stromal or chondrocyte-like states, endothelial interactions, and cell-cell communication networks (76–81). These findings suggest that endplate chondrocytes in active interface lesions should not be viewed as passive targets. Rather, they may function as local immune-responsive signaling nodes that receive macrophage cytokines, Th17-associated cytokines, NET-associated danger signals, and mast-cell mediators, while releasing chemokines, matrix fragments, and antigen-presentation-related cues that sustain immune-cell residence.

Beyond macrophages, adaptive immune imbalance may help explain why active DEBC Type A/B lesions remain unresolved. A T helper 17 (Th17)-skewed response could amplify interface inflammation through IL-17A/IL-17F signaling, particularly in the presence of IL-6, IL-1β, TNF-α, and IL-23. IL-17 can synergize with TNF-α or IFN-γ to induce inflammatory mediators and a destructive phenotype in human intervertebral disc cells (68). A similar cytokine environment may push stressed endplate chondrocytes toward chemokine production, matrix-catabolic activity, and inflammatory stress responses. In contrast, regulatory T cell (Treg)-derived IL-10 and TGF-β may restrain macrophage activation and support inflammatory resolution. An increased Th17/Treg functional ratio at the disrupted disc-endplate-marrow interface could therefore create a feed-forward loop in which endplate chondrocytes release CCL2, CXCL8/IL-8, IL-6, and matrix-degradation products, while infiltrating T cells and macrophages reinforce NF-κB, STAT3, and NLRP3 activation.

Activated neutrophils provide another plausible link between marrow inflammation and endplate destruction. Recent Modic-change bone marrow data indicate that neutrophils can acquire an activated pro-inflammatory phenotype and release neutrophil elastase capable of degrading cartilaginous endplate proteoglycans (82). Although neutrophil extracellular traps (NETs) have not yet been spatially mapped in DEBC-defined lesions, NET-associated chromatin, citrullinated histones, myeloperoxidase, neutrophil elastase, and other granular proteins could theoretically injure endplate matrix, expose matrix-derived danger signals, activate macrophages, and enhance inflammasome-related responses (83). This mechanism would connect innate immune activation with adaptive immune persistence: degraded matrix fragments and NET-associated danger signals may recruit or activate macrophages and T/NK-cell populations, thereby maintaining low-grade interface inflammation rather than allowing complete resolution.

Mast cells may also participate in this chronic lesion ecology. Mast cells have been detected in painful degenerative disc tissue, and mast-cell-conditioned media can induce inflammatory, catabolic, and pro-angiogenic gene expression in nucleus pulposus and cartilaginous endplate cells (84). More recent Modic-change work suggests that mast-cell activation through the Mrgprb2/MRGPRX2-related axis can aggravate Modic-like lesions by reshaping local immune niches, recruiting macrophages, and favoring pro-inflammatory macrophage polarization (85). In active DEBC Type A/B lesions, mast-cell degranulation products, including tryptase, chymase, histamine, TNF-α, IL-6, VEGF, and matrix-remodeling enzymes, could feed back onto endplate chondrocytes, endothelial cells, macrophages, and nociceptive fibers. This would link inflammation, angiogenesis, neurogenic sensitization, and persistent tissue remodeling at the disc–endplate–marrow interface.

Taken together, active DEBC Type A/B lesions may be sustained by an unresolved inflammatory circuit rather than by a single immune-cell lineage. Endplate disruption may expose matrix fragments, DAMPs, acidic stress, and abnormal mechanical cues, prompting local endplate chondrocytes to adopt immune-responsive and ECM-remodeling states. These cells may then interact with macrophages, T cells, neutrophils, mast cells, and endothelial cells through chemokines, cytokines, matrix-derived signals, and paracrine mediators. Within this circuit, macrophage-centered NF-κB/NLRP3 activation, Th17-skewed cytokine signaling, NET-associated danger signals, and mast-cell degranulation could reinforce one another and prevent inflammatory resolution. However, because most available single-cell and immune-mechanistic datasets have not been matched to MRI-defined DEBC Type A/B lesions, these multi-lineage links should be interpreted as testable hypotheses requiring spatial transcriptomics, multiplex immunohistochemistry, and DEBC-labeled histopathological validation.

### 5.3 Dual roles of immunity and failure of inflammatory resolution

Against this expanded adaptive-innate immune backdrop, the key biological question is not only how inflammation is initiated, but also why resolution fails after the initial interface injury. The immune response in IVDD is better understood as a balance between injury amplification and failed resolution than as a simple index of inflammatory intensity. Persistent inflammatory-cell infiltration and pro-inflammatory mediator release can promote ECM degradation, cell death, and neurovascular ingrowth. Appropriately regulated immune responses, by contrast, may help clear matrix debris, support post-injury repair signaling, and restore local homeostasis (70). Active DEBC Type A/B lesions may therefore represent an interface state in which inflammatory activation coexists with incomplete resolution. Lesions that remain chronically low-grade active may reflect ongoing immune-cell entry, inadequate inflammatory clearance, and failure of marrow responses to terminate (55).

Extracellular vesicles are another plausible route for molecular exchange across the endplate interface, but the evidence remains mostly experimental. Endplate chondrocyte-derived exosomes can regulate NF-κB signaling through the miR-133a-3p/MAML1 axis. Studies of platelet-rich plasma-derived exosomes and M1 macrophage-derived exosomes further show that vesicle signals can have divergent effects depending on the immune context (53, 54, 57). These findings support a role for paracrine communication in disc-endplate crosstalk, but they do not yet justify direct therapeutic extrapolation to active DEBC lesions.

### 5.4 Vascular-immune interactions and spatial validation

**Figure 3:**
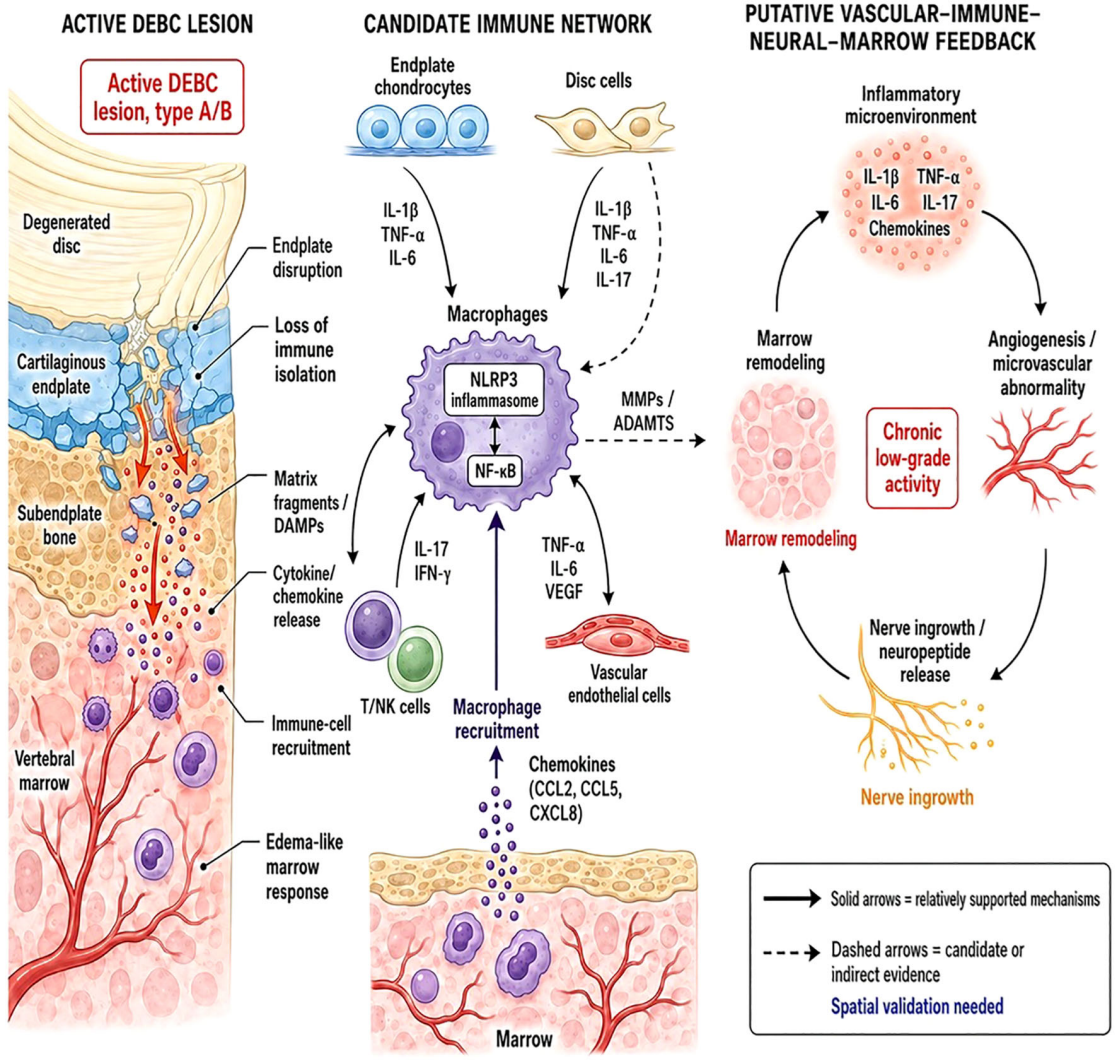
Immune and neurovascular remodeling in active DEBC lesions. Endplate disruption may weaken disc immune isolation and permit cross-interface communication through damage-associated molecular patterns, matrix fragments, cytokines, chemokines, and immune-cell recruitment. The schematic highlights a core macrophage-centered interface network involving endplate chondrocytes, disc cells, macrophages, T/NK cells, vascular endothelial cells, and marrow components. These candidate interactions may activate NF-κB and NLRP3 inflammasome pathways, amplify extracellular matrix degradation, support angiogenesis and nerve ingrowth, and maintain chronic low-grade lesion activity. Solid arrows indicate relatively supported mechanisms, whereas dashed arrows denote candidate or indirect links requiring spatial validation in DEBC-labeled lesions. Additional adaptive and innate immune components discussed in Section 5.2, including Th17/Treg imbalance, NET-associated signals, and mast-cell degranulation, are not depicted individually because their roles in DEBC-defined lesions remain hypothesis-generating and require direct spatial validation.

Vascular-immune coupling may help explain why some active DEBC lesions become chronic. Angiogenesis can provide routes for immune-cell entry and increase local exposure to circulating inflammatory mediators (86). In this setting, a vascular-immune feedback loop (right side of **Figure 3**) may reinforce chronicity: inflammatory microenvironments promote neurovascular ingrowth, neuropeptide release, and marrow responses, which can further amplify local inflammation and tissue remodeling. This inflammation-vascular-neural-marrow remodeling cycle remains to be quantified in DEBC-labeled lesions, but it provides a biologically plausible explanation for persistent low-grade activity (47, 52, 70) and its possible links to pain sensitization and functional limitation (60–66).

Spatially resolved validation is a necessary next step in moving from plausible mechanisms to DEBC-specific biology. Future studies should go beyond cross-sectional comparisons of degenerative and non-degenerative tissues. MRI-defined DEBC A/B/C/D lesions should be matched with spatial transcriptomics, immunohistochemistry, and histopathology to determine whether specific immune-cell states truly correspond to particular DEBC types. This strategy would separate direct evidence from mechanistic inference, prevent the immune microenvironment from being oversimplified as either a pain source or treatment target, and strengthen the pathological basis for clinical translation.

## 6 Translational prospects: from interface classification to clinical stratification and mechanism-guided research design

The translational value of DEBC currently lies in patient stratification, cohort enrichment, and mechanism-guided research design rather than in immediate treatment selection. For clinicians, disc desiccation, disc height loss, or the presence of Modic changes alone often fails to explain differences in pain generation or treatment response. DEBC shifts evaluation toward the lesion level by assessing endplate disruption, STIR hyperintensity in adjacent marrow, continuity between disc and subendplate marrow signals, and imaging features suggestive of edema-like activity, fatty change, or sclerotic remodeling (22–24). These imaging features should not be interpreted as direct evidence of a specific molecular pathway, pain generator, or therapeutic target. At present, DEBC is better suited to patient enrichment, subgroup analysis, and prospective mechanism testing than to stand-alone clinical decision-making.

Active DEBC Type A/B lesions should therefore be interpreted as possible interface activity, not as proof that inflammation is the pain generator. These lesions may indicate more active interface injury, endplate disruption, and marrow response, but the relationship between imaging activity, immune activity, and pain remains probabilistic rather than deterministic. Patients with chronic axial LBP, endplate-related MRI phenotypes, poor response to usual conservative care, and exclusion of obvious radiculopathy, facet-mediated pain, infection, instability, or other major confounders could be prioritized for mechanism-guided intervention studies (4, 5, 58, 59, 87–91). In this subgroup, anti-inflammatory modulation, stabilization of the endplate microenvironment, attenuation of marrow responses, and neuromodulatory interventions targeting endplate-related nociceptive pathways should be regarded as hypotheses requiring rigorous validation rather than as established DEBC-based indications.

BVNA should be interpreted not as evidence that DEBC classification guides therapy, but as an indirect proof-of-concept that interface-level imaging stratification can identify clinically responsive subgroups. Randomized controlled trials, prospective multicenter studies, and long-term follow-up data have shown that carefully selected patients with chronic axial LBP and Modic Type 1 or Type 2 endplate-related phenotypes can achieve durable improvements in pain and function after basivertebral nerve ablation (87–91). These findings are relevant to the DEBC framework because Modic Type 1 and mixed inflammatory endplate phenotypes may overlap radiologically with active DEBC Type A/B lesions, particularly when marrow edema-like signal, STIR hyperintensity, and endplate disruption coexist. The evidence should nevertheless be interpreted cautiously: BVNA trials were designed around Modic phenotypes and strict clinical selection, not around DEBC classification, and they do not establish DEBC classification as an independent treatment-selection criterion.

The caution against direct treatment extrapolation is also supported by antibiotic trials in Modic-defined cohorts: an initial randomized trial reported benefit in selected patients with chronic LBP and Modic Type 1 changes, whereas the larger AIM trial did not support routine antibiotic treatment for chronic LBP with Modic changes (92, 93). This discrepancy reinforces that endplate-related MRI phenotypes require careful clinical, biological, and trial-specific validation before they can be used as treatment-selection markers.

The main translational lesson from BVNA is therefore not that DEBC already selects neuromodulatory intervention, but that interface-centered phenotyping can reduce clinical heterogeneity. A dedicated DEBC framework could refine future trial enrichment by prospectively combining Modic status with DEBC type, STIR-defined lesion activity, endplate defect severity, lesion continuity, axial pain phenotype, symptom duration, functional limitation, and standardized exclusion of radiculopathy, facet-mediated pain, infection, instability, or other competing pain generators. Such a strategy may help determine whether active DEBC Type A/B lesions identify a more homogeneous interface-driven subgroup for neuromodulatory trials. Until prospective DEBC-stratified data are available, BVNA should be discussed as retrospective support for the value of interface-level stratification rather than as proof that DEBC classification independently predicts treatment response.

Low-activity or sclerotic DEBC Type C/D lesions raise different translational questions from Type A/B lesions. Type C usually suggests fatty change or a relatively low-activity state, whereas Type D more closely resembles sclerosis, chronic structural remodeling, or post-inflammatory repair (22, 24). In these lesions, anti-inflammatory therapy should not be assumed to be the main strategy. Subendplate bone stiffness, impaired nutrient transport, reduced disc load-bearing capacity, and an unfavorable regenerative microenvironment may be more important determinants of long-term treatment response (16, 17, 39, 44). Type C/D lesions may therefore be more informative for studying structural remodeling, mechanical environment adjustment, regenerative failure risk, and surgical decision support.

DEBC may also help explain why biologic and regenerative therapies produce inconsistent results across patients. Intradiscal cell therapy, extracellular vesicles, and biomaterials are most likely to perform consistently when endplate transport, local inflammation, and mechanical conditions remain permissive (67, 94–96). If transport failure, marrow inflammation, abnormal mechanical loading, or acidic metabolism is not recognized or corrected, an intradiscal injection alone may not provide the interface conditions needed for cell survival, matrix synthesis, and nutrient exchange (17, 44, 47). DEBC is therefore better used as an imaging screening variable and failure-risk tool before biologic therapy than as a direct indication for regeneration.

Clinical trials in IVDD should use DEBC to reduce heterogeneity rather than to replace clinical judgment. Future studies should not enroll patients solely on Pfirrmann grade, disc height loss, or nonspecific degenerative findings. Stratification should incorporate DEBC type, Modic phenotype, STIR-defined lesion activity, endplate defect severity, lesion continuity, pain duration, functional scales, psychosocial factors, neurological findings, and exclusion of competing pain generators (22, 23, 87–91). This design may reduce dilution of treatment effects in heterogeneous cohorts and help test whether specific interface phenotypes predict response to anti-inflammatory modulation, neuromodulation, regenerative repair, or structural stabilization. In current practice, however, DEBC should be interpreted alongside the full clinical phenotype and should not be used as an independent treatment indication or as proof of treatment responsiveness.

The clinical use of DEBC should therefore begin from phenotype enrichment rather than pain attribution. A patient with an active DEBC Type A/B lesion may have an interface phenotype that is biologically plausible and relevant for study selection, but this does not prove that the lesion is the dominant source of symptoms. Conversely, the absence of an active DEBC lesion does not exclude clinically meaningful pain from annular, neural, facet-mediated, or centrally sensitized mechanisms. DEBC-based interpretation should therefore be combined with clinical examination, pain phenotype, functional assessment, psychosocial context, and exclusion of competing pain generators before being used in research stratification or trial design.

## 7 Current evidence gaps and future directions

The current evidence base supports DEBC as a complementary lesion-level imaging framework, but not yet as a validated pathological or therapeutic classifier. Available studies come from imaging classification, cross-sectional clinical observation, animal modeling, basic experiments, and omics analyses. They differ in study population, spinal region, sampling site, imaging protocol, and validation strategy. Integrating DEBC classification with endplate-marrow crosstalk and immune remodeling is theoretically well grounded, but its mechanistic correspondence and clinical predictive value remain to be established (18, 22, 24). The main evidence domains supporting this synthesis, together with their calibrated inferential distance from DEBC-specific claims, are summarized in **Table 3**.

**Table 3:** Evidence level based on directness and validation status across evidence domains supporting the DEBC framework.

Representative studies	Evidence domain	Contribution to the DEBC framework	Major limitations	Evidence level based on directness and validation status
Rajasekaran 2025 (22); Jagadish 2025 (24); Esposito 2025 (23)	Imaging classification	Supports lesion-level assessment of the disc, endplate, and adjacent marrow as an integrated imaging complex and indicates complementarity with existing MRI grading systems.	Mostly cross-sectional or descriptive evidence; limited longitudinal pathological matching, treatment-response validation, and multicenter reproducibility data.	Moderate. Directly DEBC-relevant, but limited by cross-sectional design and insufficient pathological or longitudinal validation.
Feng 2025 (25); Feng 2021 (26); Lagerstrand 2021 (27); Chen 2024 (28)	Endplate-Modic relationship	Supports the association of cartilaginous endplate injury, avulsion, defects, or inflammatory pain-axis activation with Modic-like marrow changes and symptom-related interface pathology.	Mostly Modic- or endplate-focused rather than DEBC-labeled; causal sequence, lesion continuity, and subtype specificity remain insufficiently validated.	Moderate. Supports endplate-centered pathology, but remains indirect for DEBC-specific classification.
Urban 2004 (8); Ashinsky 2020 (16); Sun 2024 (17); Crump 2023 (37)	Endplate transport and microvascular biology	Explains why endplate nutrient transport, microvascular supply, diffusion capacity, and metabolic barrier function are biologically relevant to disc-endplate-marrow coupling.	Direct correspondence between DEBC type and transport or microvascular status has not been established; most studies do not use MRI-defined DEBC phenotypes.	Moderate. Robust biological basis, but DEBC subtype mapping remains indirect.
Li 2025 (33); Peng 2025 (34); Dong 2025 (35); Zhao 2021 (42)	Mechanical and metabolic mechanisms	Supports candidate mechanisms linking mechanical overload, Piezo1 signaling, IFT88/TRPV4-related mechanobiology, acid-sensing pathways, ROS production, inflammasome activation, and endplate or disc-cell stress.	Evidence is mainly experimental or mechanistic; direct validation in human MRI-defined DEBC lesions is lacking.	Low. Mechanistically plausible, but not mapped to DEBC-labeled human lesions.
Wang 2024 (18); Ren 2025 (70); Shi 2024 (56); Tu 2022 (72); Liang 2025 (74)	Immune and omics evidence	Supports involvement of OPN/NF-kappaB/NLRP3 signaling, macrophage recruitment, inflammation-associated endplate cell states, and candidate cell-cell communication networks.	Most samples are not obtained from DEBC-labeled lesions; spatial validation across paired disc, endplate, and marrow tissues remains insufficient.	Low. Biologically important and hypothesis-generating, but DEBC directness and spatial validation are limited.
Khalil 2019/2024 (87, 90); Fischgrund 2019/2020 (88, 89); Zhou 2024 (95); Zhang 2025 (96)	Translational stratification and treatment-response inference	BVNA and regenerative-therapy studies suggest that interface-related imaging phenotypes may enrich clinical cohorts and reduce heterogeneity in trial design.	Most evidence is based on Modic phenotypes, general IVDD stratification, or treatment-specific cohorts; DEBC has not been prospectively validated as a predictor of efficacy.	Very low. Retrospective proof-of-concept only; no prospective DEBC-stratified efficacy validation.

Evidence levels were calibrated narratively rather than by formal GRADE assessment. Ratings were based on five predefined parameters: directness to MRI-defined DEBC classification; study design and reproducibility; human relevance and sample scale; availability of longitudinal, histopathological, molecular, or clinical validation; and translational linkage to patient stratification or treatment-response prediction. “High” was reserved for evidence directly derived from DEBC-labeled human lesions with reproducible longitudinal, pathological, or clinical validation. “Moderate” indicates evidence that supports a major component of the DEBC framework but remains limited by cross-sectional design, incomplete pathological matching, or indirect validation. “Low” indicates biologically plausible mechanistic or omics evidence that is mostly experimental, indirect, or not obtained from DEBC-labeled lesions. “Very low” indicates exploratory translational inference without prospective DEBC-stratified validation. These levels indicate inferential distance from DEBC-specific claims and should not be interpreted as a formal systematic-review evidence grade.

Because the current evidence spans imaging classification, clinical observation, animal modeling, mechanistic experiments, and omics studies, **Table 3** uses a narrative evidence-calibration approach rather than a formal GRADE assessment. The evidence level was calibrated according to five predefined parameters: directness to MRI-defined DEBC classification; study design and reproducibility; human relevance and sample scale; availability of longitudinal, histopathological, molecular, or clinical validation; and translational linkage to patient stratification or treatment-response prediction. “High” indicates evidence that is directly relevant to the DEBC framework, reproducible, human-based, and supported by longitudinal, pathological, or clinical validation. “Moderate” indicates evidence that supports a major component of the DEBC framework but remains limited by cross-sectional design, incomplete pathological matching, or indirect clinical validation. “Low” indicates mechanistic or omics evidence that is biologically plausible but mostly indirect, experimental, or not derived from DEBC-labeled lesions. “Very low” indicates exploratory translational inference without prospective DEBC-stratified validation. These levels are intended to clarify inferential distance rather than to rank study quality in a systematic-review sense.

### 7.1 Longitudinal imaging-clinical validation

Longitudinal imaging-clinical cohorts remain a central missing evidence domain. Current DEBC-related clinical data are mostly cross-sectional or observational, which makes it difficult to determine the temporal sequence linking endplate injury, marrow change, immune remodeling, DEBC phenotype, and pain (22, 24). Future studies should follow DEBC Type transitions, progression of endplate defects, pain intensity, functional limitation, treatment response, and recurrence risk over time. Establishing temporal order is essential for determining whether DEBC Type marks degenerative progression, identifies a pain-associated phenotype, or stratifies treatment response.

### 7.2 Standardized interpretation and multicenter reproducibility

Standardized interpretation is required before DEBC can become a reliable stratification tool. At present, the framework is conceptually attractive, but its clinical utility will depend on whether different readers can assign DEBC types consistently and whether the same lesion is interpreted similarly across scanners, institutions, and spinal regions. Future studies should therefore report intraobserver and interobserver agreement for DEBC type assignment, preferably using weighted kappa statistics for categorical grading and intraclass correlation coefficients for quantitative or semi-quantitative imaging features. Reader expertise should also be documented, because agreement may differ between musculoskeletal radiologists, spine surgeons, general radiologists, and trained research readers.

Several imaging components require explicit operational definitions. STIR hyperintensity should be reported as STIR-defined lesion activity or edema-like marrow response rather than as direct proof of inflammation. Future protocols should specify whether STIR activity is assessed qualitatively, semi-quantitatively, or by signal-intensity ratios relative to adjacent normal marrow or other predefined reference tissues. Endplate defects should also be standardized by location, size, depth, continuity, and whether they involve the cartilaginous endplate, bony endplate, or both. Lesion continuity should be defined by the spatial relationship among disc signal abnormality, endplate disruption, and adjacent marrow response on matched sagittal and axial images. Similarly, marrow edema-like change, fatty change, sclerosis, disc signal loss, disc height reduction, and endplate irregularity should be recorded using prespecified criteria rather than relying solely on global visual impression.

Multicenter reproducibility is particularly important because DEBC assessment may be sensitive to MRI acquisition parameters. Field strength, slice thickness, sequence orientation, fat-suppression method, STIR inversion time, image resolution, coil configuration, and post-processing can all influence the visibility of endplate defects and marrow signal changes. Future studies should therefore harmonize minimum MRI protocols, define acceptable sequence parameters, and report whether DEBC grading was performed using local readings, central readings, or adjudicated consensus. A reader-training atlas with representative DEBC Type A/B/C/D examples, borderline cases, and common mimics would help reduce interpretive drift across centers.

External validation should also test whether DEBC adds information beyond established grading systems. Future multicenter cohorts should evaluate the incremental value of DEBC over Modic classification, Pfirrmann grading, endplate defect scores, and conventional clinical variables. Reproducibility should be examined not only in lumbar lesions but also across cervical and thoracic regions, where anatomy, motion patterns, marrow composition, and image quality differ. Only after prospective evidence shows acceptable reader agreement, protocol robustness, and added prognostic or stratification value can DEBC be responsibly used for trial enrichment or mechanism-guided clinical research.

### 7.3 Imaging-omics integration and lesion-targeted validation

Imaging-omics integration offers the clearest path for testing whether DEBC types correspond to distinct cellular programs. Single-cell RNA sequencing and single-nucleus RNA sequencing have revealed cellular heterogeneity, progenitor-like states, inflammation-associated cell states, immune-cell populations, and intercellular communication networks in degenerative disc, annulus fibrosus, nucleus pulposus, and endplate tissues (71–75, 97, 98). Paired-sample and tissue-heterogeneity studies further show why conventional histology and bulk transcriptomics often fail to explain differences in progression rate, inflammatory activity, and pain manifestation between adjacent lesions (75–79). Recent single-nucleus and spatial transcriptomic work in experimental disc degeneration has also begun to map spatial cell subpopulations and regional cellular dynamics within degenerative regions, providing a technical reference for future spatial validation of DEBC-labeled lesions (74).

A useful working hypothesis is that each DEBC type reflects a different lesion ecology, but this remains unproven. Type A/B lesions may be enriched for inflammatory immune cells, activated endplate chondrocyte states, macrophage–chondrocyte communication, fibrovascular remodeling, and incomplete inflammatory resolution. Type C lesions may align more closely with lipid-metabolic change, fatty marrow replacement, and low-grade chronic remodeling, whereas Type D lesions may involve osteogenic, fibrotic, and sclerotic subendplate bone-remodeling programs (56, 72, 74, 80, 81, 97, 98). However, these associations are still largely inferred from imaging–mechanism reasoning and broader IVDD or Modic-related evidence. They should not be interpreted as validated molecular signatures of DEBC types without direct matching of imaging phenotype, histopathology, and spatial molecular data within the same lesion.

Future studies should therefore begin with MRI-defined DEBC lesions and preserve the link between imaging phenotype and tissue biology. Preoperative MRI can define DEBC type, STIR-defined lesion activity, endplate defect location, lesion continuity, and the approximate boundary of the disc-endplate-marrow interface lesion. Tissue sampling should then be anchored to that region, with paired cartilaginous endplate and subendplate marrow samples, and adjacent disc tissue when available, obtained from the same MRI-characterized lesion during clinically indicated procedures or, in carefully justified research settings, through CT- or ultrasound-guided core biopsy when feasibility, safety, tissue yield, and spatial precision can be ensured. The essential requirement is not a single fixed protocol, but maintained spatial correspondence between the MRI-defined lesion and the sampled interface tissues.

Lesion-matched tissue analysis should combine complementary spatial and molecular methods. Histopathology can define structural endplate disruption, marrow remodeling, fibrosis, sclerosis, vascularity, and inflammatory infiltrates; spatial transcriptomics can map regional cellular programs across the disc-endplate-marrow interface; single-nucleus RNA sequencing can resolve cell states in mineralized or difficult-to-dissociate tissues; and multiplex immunohistochemistry or immunofluorescence can validate the spatial proximity of endplate chondrocytes, macrophages, T cells, neutrophils, mast cells, endothelial cells, and nerve fibers. This combined approach would allow active Type A/B lesions to be tested for macrophage-enriched niches, Th17/Treg-associated signaling, NET-associated signals, mast-cell activation, endothelial activation, nerve ingrowth, and inflammatory endplate chondrocyte states, while Type C/D lesions could be examined for adipogenic, osteogenic, fibrotic, senescent, or sclerotic remodeling programs.

At present, this imaging-to-molecular mapping should be considered a research agenda rather than an established diagnostic pathway. Key challenges include small tissue volume, difficulty harvesting intact cartilaginous endplate, contamination between disc and marrow compartments, sampling bias, RNA degradation, decalcification-related molecular loss, limited access to asymptomatic controls, and ethical constraints on biopsy in patients without surgical indication. Nevertheless, MRI-targeted paired sampling of subendplate marrow and cartilaginous endplate remains the most direct strategy for separating DEBC-specific biology from indirect inference. Only such lesion-matched validation can determine whether the biological interpretations proposed in **Table 1** reflect true cellular states and molecular programs, or whether they remain useful but non-specific imaging hypotheses.

The evidentiary boundaries of the DEBC framework are summarized in **Box 1** to separate established imaging observations from indirect mechanistic inference and hypotheses requiring lesion-matched validation.

Box 1 **Evidence boundaries of the DEBC framework**

Established observations. Modic classification is a widely used MRI framework for describing vertebral marrow signal changes adjacent to the endplate. DEBC provides a complementary lesion-level imaging approach by integrating disc signal, endplate integrity, adjacent marrow response, lesion continuity, and STIR-defined activity. Endplate injury, impaired transport, and marrow signal abnormalities are established observations relevant to interface-centered degeneration.

Indirect inference. The proposed biological meanings of DEBC Type A/B/C/D lesions are inferred from broader evidence on Modic changes, endplate defects, IVDD pathology, disc nutrition, mechanobiology, immune remodeling, and omics studies. STIR hyperintensity is suggestive of edema-like lesion activity, but it does not directly prove inflammation, pain generation, or a specific molecular program. Overlap between Modic Type 1 and active DEBC Type A/B lesions does not make the two classifications equivalent.

Testable hypotheses. Active DEBC Type A/B lesions may represent unresolved interface inflammation involving macrophage-centered signaling, Th17/Treg imbalance, NET-associated danger signals, mast-cell degranulation, vascular-immune coupling, and immune-responsive endplate chondrocytes. Type C/D lesions may be more closely related to fatty change, sclerosis, fibrosis, altered transport, or chronic structural remodeling. These interpretations remain hypotheses rather than validated DEBC-specific cellular states.

Required validation. Future studies should directly match MRI-defined DEBC lesions with paired cartilaginous endplate and subendplate marrow sampling, histopathology, spatial transcriptomics, single-nucleus RNA sequencing, multiplex immunohistochemistry, reader-reliability analysis, longitudinal imaging, and clinical outcome data. Only such validation can determine whether DEBC types correspond to reproducible molecular lesion states, pain-associated phenotypes, or treatment-response patterns.

### 7.4 Clinical translation limitations

The main translational risk is to overread DEBC as a pain diagnosis. Pain generation reflects peripheral nociceptive input, neurovascular ingrowth, basivertebral nerve-related signaling, radiculopathy, facet or sacroiliac pathology, psychosocial factors, and peripheral or central sensitization. DEBC is therefore more defensible as a stratification variable, trial-enrichment tool, and entry point for mechanistic validation than as an independent diagnostic standard or direct treatment indication. A DEBC lesion may be biologically plausible and clinically relevant in selected patients, but it should not be treated as a stand-alone pain generator without clinical correlation and exclusion of competing sources. Only when DEBC types show reproducible links to clinical outcomes, cellular ecologies, pathological features, pain phenotypes, and treatment response will they be ready for use in precision pathways for IVDD.

## 8 Conclusions

IVDD is increasingly understood as an interface-related process involving the disc, cartilaginous endplate, and adjacent vertebral marrow, rather than as degeneration confined to the disc alone. Endplate injury, calcification, and microfracture may disrupt nutrient transport and barrier function, while microvascular alteration, abnormal mechanics, inflammatory mediator exchange, and marrow remodeling may contribute to cross-tissue progression. These mechanisms provide a biologically plausible basis for interpreting interface-centered imaging phenotypes, but they should not be regarded as proven pathological substrates for individual DEBC types.

DEBC provides a complementary imaging entry point into this cross-tissue process. Compared with conventional Modic classification, DEBC may broaden lesion-level description by integrating disc signal, endplate integrity, adjacent marrow response, lesion continuity, and STIR-defined activity. However, this does not mean that DEBC replaces Modic classification or has already demonstrated a validated interpretive advantage over existing MRI frameworks. Its added value remains to be tested against established MRI grading systems, clinical phenotypes, reader reliability, longitudinal outcomes, and tissue-level validation. STIR hyperintensity should also be interpreted cautiously as an imaging feature suggestive of edema-like lesion activity, not as direct proof of inflammation, pain generation, or a specific molecular program.

The current value of DEBC lies in hypothesis generation, lesion-level stratification, and research enrichment rather than in stand-alone diagnosis or treatment selection. If future studies show reproducible links between DEBC types, histopathological features, spatial cellular ecologies, molecular programs, pain phenotypes, and treatment-response patterns, DEBC may help refine patient stratification and mechanism-guided trial design in IVDD. Until such validation is available, DEBC should be interpreted as a complementary research framework that may extend, but should not replace, existing Modic- and degeneration-based imaging classifications.
